# Oral administration of trace element magnesium significantly improving the cognition and locomotion in hepatic encephalopathy rats

**DOI:** 10.1038/s41598-017-02101-8

**Published:** 2017-05-12

**Authors:** Ying Li, Chang Xue Ji, Li Hong Mei, Jin Wei Qiang, Shuai Ju

**Affiliations:** 1 Department of Radiology, Jinshan Hospital, Fudan University, Shanghai, 201508 China; 2 Department of Dermatology, Jinshan Hospital, Fudan University, Shanghai, 201508 China

## Abstract

The therapeutic effects of iron, zinc and magnesium trace elements, as well as rifaximin were investigated and compared in HE rats. In this study, HE rats were treated with either ferrous sulfate (HE-Fe, 30 mg/kg/day), zinc sulfate (HE-Zn, 30 mg/kg/day), magnesium sulfate (HE-Mg, 50 mg/kg/day) or rifaximin (HE-Rf, 50 mg/kg/day), which was mixed with water and administered orally for 61 days. The Morris water maze (MWM) and open-field tests were used to evaluate cognitive and locomotor function. The blood ammonia levels before and after administration of the glutamine challenge test, manganese concentration and glutamine synthetase (GS) activity were measured. Significantly longer MWM escape latencies, less locomotor activity, higher blood ammonia levels, higher brain manganese concentrations and higher GS activity were observed in HE rats. However, HE-Mg and HE-Rf rats had significantly shorter MWM escape latencies, increased locomotor activity, lower blood ammonia, lower brain manganese concentrations and lower GS activity. Partial improvements were observed in HE-Fe and HE-Zn rats. The results indicated that oral administration of magnesium can significantly improve the cognitive and locomotor functions in HE rats by reducing the brain manganese concentration and regulating GS activity.

## Introduction

Hepatic encephalopathy (HE) is a major complication of cirrhosis and (or) portal-systemic shunting^1^. Its pathogenesis is not completely clear. It has been suggested that nitrogen metabolism dysfunction may cause neurological symptoms^2^. HE significantly affects the quality of daily life and may ultimately lead to death; however, HE can be reversed with the appropriate treatment^1, 2^.

HE patients are known to have higher levels of ammonia in their blood. Thus, current HE treatments focus on non-absorbable disaccharides (e.g., lactulose) or antibiotics (e.g., rifaximin) to reduce ammoniagenesis in the intestines and lower blood ammonia levels^3^.

Recent studies showed that trace element concentrations varied in cirrhotic patients with HE^4^. Lower serum zinc, magnesium and iron but higher serum manganese and copper were reported in cirrhotic patients^5–7^. Zinc, magnesium, and iron are co-enzymes of a variety of metabolic enzymes. Deficiencies in zinc, magnesium and iron levels may lead to metabolic disorders and negatively impact behavior and cognition^8–10^. On the other hand, high manganese levels can be neurotoxic. Excessive levels of manganese lead to cognitive impairments and extrapyramidal symptoms^11^.

Oral administration of zinc effectively improves HE symptoms^12^. However, whether oral administration of iron or magnesium is also beneficial is not known. Previous studies demonstrated that trace elements, such as iron and magnesium, can influence manganese absorption in the intestines^13, 14^. We hypothesize that iron and magnesium supplementation may improve HE by reducing intestinal manganese absorption and subsequently reduce brain manganese concentrations. The aim of this study was to investigate and compare the therapeutic effects of iron, zinc, and magnesium trace elements as well as rifaximin in HE rats.

## Results

### Effects on general state

There were no significant differences in body weight, food consumption or defecation between the groups at any time following the partial portal vein ligation. However, from the end of the 4th week (day 28) to 8th week (day 56) HE-Zn rats consumed less water (*P* = 0.013, *P* = 0.015, and *P* < 0.001, respectively) (Supplementary Tables 1–4).

### Effects on cognition and locomotion

**Figure 1:**
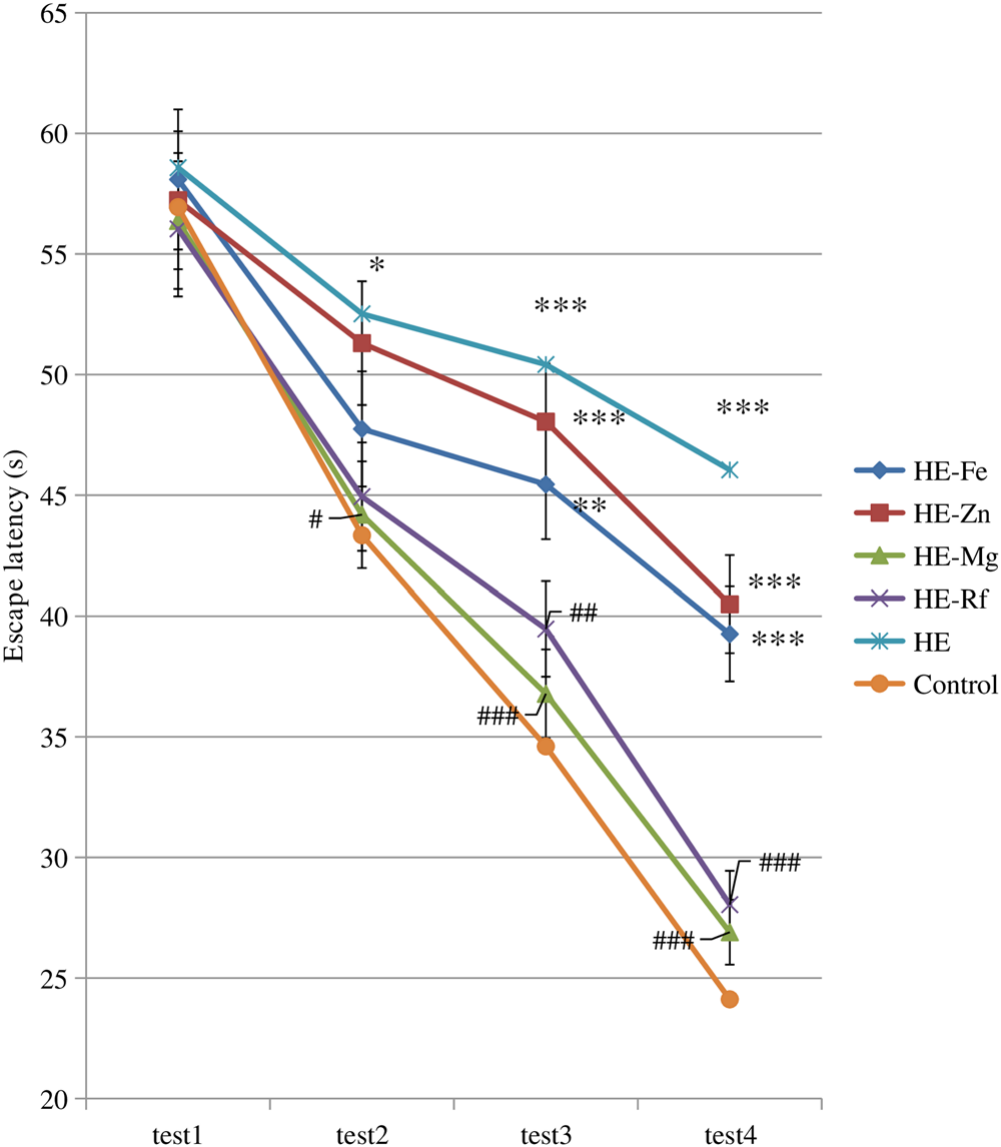
The escape latencies of four test trials of the Morris water maze in each group. The escape latencies of control rats gradually decreased as training time increased. Compared to controls, HE rats had significantly longer escape latencies during the 2nd, 3rd and 4th tests (*P* = 0.021, *P* < 0.001, and *P* < 0.001, respectively); HE-Fe and HE-Zn rats from the 3rd to 4th tests (*P* = 0.006 and *P* < 0.001; *P* < 0.001 and *P* < 0.001, respectively). Compared to HE rats, HE-Mg rats had significantly shorter escape latencies during the 2nd, 3rd and 4th tests (*P* = 0.027, *P* < 0.001, and *P* < 0.001, respectively). HE-Rf rats had significantly longer escape latencies during the 3rd and 4th tests (*P* = 0.005 and *P* < 0.001, respectively). Eight rats in each group, *P < 0.05, **P < 0.01, ***P < 0.001, control rat comparison; ^#^ *P* < 0.05, ^##^ *P* < 0.01, ^###^ *P* < 0.001, HE rat comparison. The *P* value is generated using One way ANOVA with a Bonferroni correction.

The escape latencies of control rats decreased gradually as training time increased. Relative to controls, HE rats had significantly longer escape latencies during the 2nd, 3rd and 4th tests (*P* = 0.021, *P* < 0.001, and *P* < 0.001, respectively). Relative to controls, HE-Fe rats (*P* = 0.006 and *P* < 0.001) and HE-Zn rats (*P* < 0.001 and *P* < 0.001) had significantly longer escape latencie at the 3rd and 4th tests. Compared to HE rats, HE-Mg rats had significantly shorter escape latencies during the 2nd, 3rd and 4th tests (*P* = 0.027, *P* < 0.001 and *P* < 0.001, respectively). HE-Rf rats had significantly shorter escape latencies during the 3rd and 4th tests (*P* = 0.005 and *P* < 0.001, respectively). The escape latencies did not differ between the HE-Mg, HE-Rf and control groups (both *P* > 0.05). (Fig. 1, Supplementary Table 5.1).

**Figure 2:**
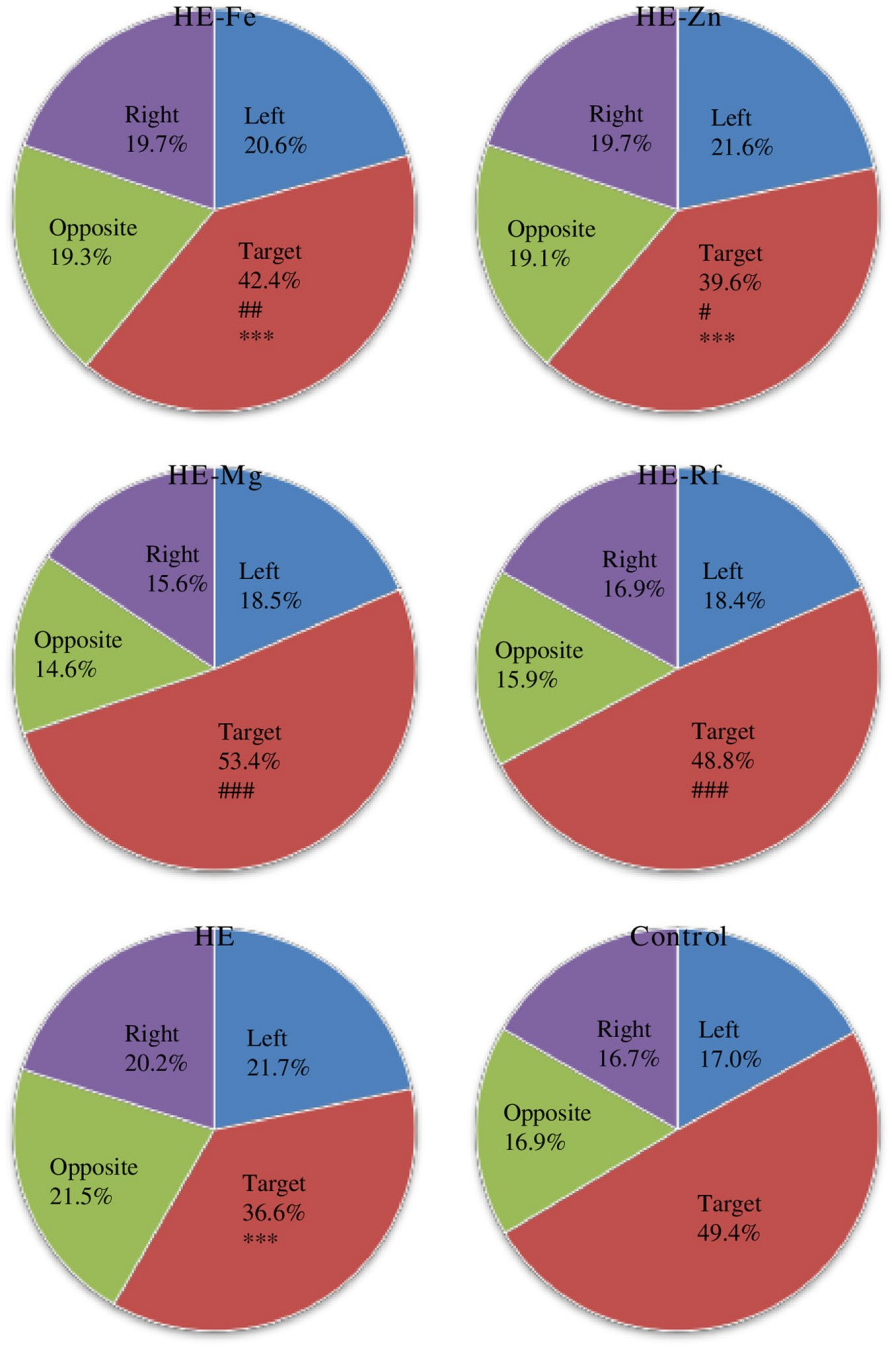
The percentage of time spent in different quadrants of the water maze for each group (the total time: 60 seconds). The target quadrant is the region where the escape platform was placed previously. The percentage of time spent in the target quadrant is significantly smaller in the HE-Fe (P < 0.001), HE-Zn (P < 0.001), and HE rats (P < 0.001) group when compared to the control. However, HE-Fe, HE-Zn, HE-Mg and HE-Rf rats spent more time in the target quadrant when compared to HE rats (P = 0.007, P = 0.038, P < 0.001 and P < 0.001, respectively). Eight rats in each group, *P < 0.05, **P < 0.01, ***P < 0.001 control rat comparison, ^#^ *P* < 0.05, ^##^ *P* < 0.01, ^###^ *P* < 0.001, HE rat comparison. The *P* value is generated using One way ANOVA with a Bonferroni correction.

After the escape platform was removed, HE-Fe, HE-Zn and HE rats spent less time in the target quadrant (former escape platform area) relative to controls (*P* < 0.001, *P* < 0.001, and *P* < 0.001, respectively). HE-Fe, HE-Zn, HE-Mg and HE-Rf rats spent more time in the target quadrant relative to HE rats (*P* = 0.007, *P* = 0.038, *P* < 0.001 and *P* < 0.001, respectively). There were no differences in the percentages of time spent in the target quadrant between HE-Mg, HE-Rf and control rats (both *P* > 0.05) (Fig. 2, Supplementary Table 5.2).

**Figure 3:**
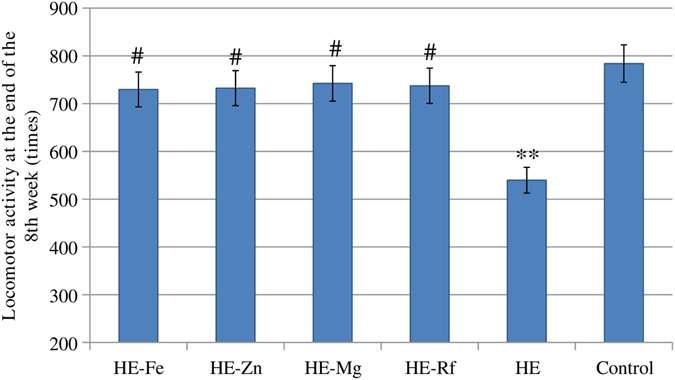
The locomotor activity at the end of the 8th week in each group. No significant differences in locomotor activity were found in HE-Fe rats, HE-Zn rats, HE-Mg rats and HE-Rf rats when compared to controls. HE rats had significantly decreased locomotor activity (*P* = 0.007). Significant increases in locomotor activity were found in HE-Fe rats (*P* = 0.033), HE-Zn rats (*P* = 0.038), HE-Mg rats (*P* = 0.027), and Rf rats (*P* = 0.043) when compared to HE rats. Eight rats in each group, *P < 0.05, **P < 0.01, ***P < 0.001, control rat comparison, ^#^ *P* < 0.05, ^##^ *P* < 0.01, ^###^ *P* < 0.001, HE rat comparison. The *P* value is generated using One way ANOVA with a Bonferroni correction.

There were no significant differences in locomotor activity between controls and HE-Fe rats, HE-Zn rats, HE-Mg rats and Rf rats. He rats had significantly decreased locomotor activity (*P* = 0.007). Significantly increased locomotor activity was found in HE-Fe rats (*P* = 0.033), HE-Zn rats (*P* = 0.038), HE-Mg rats (*P* = 0.027), and Rf rats (*P* = 0.043) relative to HE rats (Fig. 3, Supplementary Table 6).

### Blood ammonia, trace elements and GS activity in the brain

**Figure 4:**
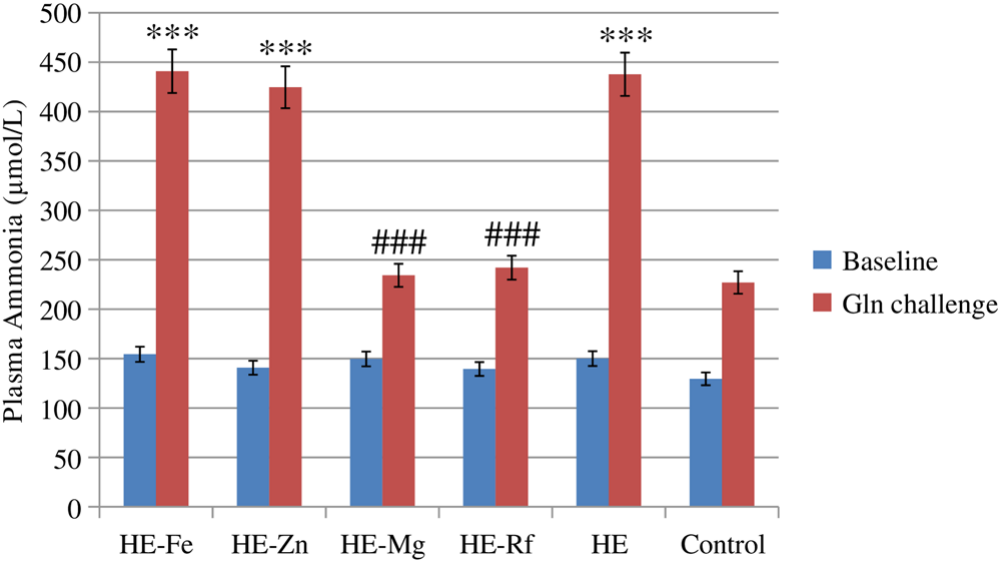
Fasting blood ammonia levels at the end of the 8th week before and after glutamate challenge in each group. Before glutamate challenge, there were no significant differences in blood ammonia levels among the groups. Thirty minutes after oral administration of glutamate, HE-Fe, HE-Zn and HE rat exhibited significantly higher blood ammonia levels when compared to controls (all *P* < 0.001). Blood ammonia levels were significantly lower in HE-Mg rats and HE-Rf rats when compared to HE rats (both *P* < 0.001); there were no differences between HE-Mg rats, HE-Rf rats and control rats (both *P* > 0.05). Eight rats in each group, *P < 0.05, **P < 0.01, ***P < 0.001, control rat comparison, ^#^ *P* < 0.05, ^##^ *P* < 0.01, ^###^ *P* < 0.001, HE rat comparison. The *P* value is generated using One way ANOVA with a Bonferroni correction.

At the end of the 8th week, there were no significant differences in fasting blood ammonia levels between the groups. However, 30 minutes after oral administration of glutamate, significantly higher blood ammonia levels were found in HE-Fe, HE-Zn and HE rat when compared to controls (all *P* < 0.001). Blood ammonia was significantly lower in HE-Mg rats and HE-Rf rats relative to HE rats (both *P* < 0.001). There were no differences in blood ammonia levels between HE-Mg rats, HE-Rf rats and HE rats (both *P* > 0.05) (Fig. 4, Supplementary Table 7).

There were no significant differences between the groups in calcium, magnesium, and ferrum concentrations in the basal ganglia. HE-Fe, HE-Rf and HE rats had higher copper concentrations in the basal ganglia when compared to controls (*P* = 0.047, *P* = 0.019, and *P* = 0.033, respectively). Zinc concentrations in the basal ganglia was lower in HE-Rf and HE rats relative to controls (*P* = 0.023, *P* = 0.036, respectively), and higher in HE-Zn rats when compared to HE rats (*P* = 0.048). Manganese concentrations in the basal ganglia were higher in HE-Rf rats and HE rats when compared to controls (both *P* < 0.001) and lower in HE-Fe rats, HE-Zn rats and HE-Mg rats when compared to HE rats (all *P* < 0.001) (Table 1).

**Table 1:** Trace element concentrations in the basal ganglia across groups.

Basal ganglia	Ca (mg/g)	Mg (mg/g)	Fe (μg/g)	Cu (μg/g)	Zn (μg/g)	Mn (μg/g)
HE-Fe	0.48 ± 0.19	0.23 ± 0.1	12.9 ± 2.91	5.12 ± 0.82*	47.75 ± 11.42	1.06 ± 0.12^###^
HE-Zn	0.47 ± 0.18	0.24 ± 0.12	10.09 ± 2.79	4.95 ± 1.55	55.97 ± 12.86^#^	1.04 ± 0.24^###^
HE-Mg	0.51 ± 0.26	0.27 ± 0.13	9.88 ± 3.2	4.75 ± 1.33	52.38 ± 18.89	0.86 ± 0.19^###^
HE-Rf	0.43 ± 0.14	0.21 ± 0.11	10.11 ± 3.56	5.74 ± 1.26*	35.47 ± 15.34*	1.59 ± 0.03***
HE	0.44 ± 0.22	0.2 ± 0.07	11.16 ± 5.35	5.84 ± 1.63*	36.75 ± 16.4*	1.6 ± 0.04***
Control	0.45 ± 0.17	0.27 ± 0.11	9.75 ± 2.13	3.89 ± 1.05	56.69 ± 11.83	0.85 ± 0.24

Six rats in each group, *P < 0.05, **P < 0.01, ***P < 0.001, control rat comparison, ^#^ *P* < 0.05, ^##^ *P* < 0.01, ^###^ *P* < 0.001, HE rat comparison. The *P* value is generated using One way ANOVA with a Bonferroni correction.

There were no significant differences in calcium, magnesium, ferrum, copper, and zinc concentrations in the cortex between groups. Cortical manganese concentration was higher in HE-Zn, HE-Rf and HE rats relative to controls (*P* = 0.041, *P* = 0.026, and *P* = 0.035, respectively) and lower in HE-Mg rats when compared to HE rats (*P* = 0.049) (Table 2).

**Table 2:** Trace element concentrations in the cortex across groups.

Cortex	Ca (mg/g)	Mg (mg/g)	Fe (μg/g)	Cu (μg/g)	Zn (μg/g)	Mn (μg/g)
HE-Fe	0.41 ± 0.14	0.25 ± 0.1	6.01 ± 2.06	1.36 ± 0.7	19.82 ± 8.62	0.75 ± 0.28
HE-Zn	0.35 ± 0.12	0.23 ± 0.15	5.9 ± 2.07	1.31 ± 0.49	22.45 ± 7.89	0.76 ± 0.26*
HE-Mg	0.47 ± 0.2	0.28 ± 0.12	6.57 ± 2.3	1.15 ± 0.36	20.06 ± 9.57	0.5 ± 0.17^#^
HE-Rf	0.3 ± 0.22	0.34 ± 0.28	6.95 ± 2.86	1.53 ± 0.63	21.45 ± 7.41	0.78 ± 0.24*
HE	0.27 ± 0.14	0.23 ± 0.13	7.15 ± 3.14	1.49 ± 0.43	16.75 ± 3.26	0.85 ± 0.35*
Control	0.38 ± 0.18	0.24 ± 0.12	6.87 ± 1.93	1.19 ± 0.64	22.24 ± 8.34	0.48 ± 0.14

Six rats in each group, *P < 0.05, **P < 0.01, ***P < 0.001, control rat comparison, ^#^ *P* < 0.05, ^##^ *P* < 0.01, ^###^ *P* < 0.001, HE rat comparison. The *P* value is generated using One way ANOVA with a Bonferroni correction.

Compared to controls, HE-Mg rats had higher fecal magnesium levels (*P* < 0.001). Fecal iron levels were higher in HE-Fe, HE-Rf and HE rats (*P* < 0.001, *P* = 0.044, and *P* = 0.022, respectively) relative to controls. Fecal zinc levels in HE-Zn, HE-Rf and HE rats were higher relative to controls (*P* < 0.001, *P* = 0.017, and *P* = 0.028, respectively). HE-Mg rats had higher fecal manganese levels (*P* = 0.009). The fecal manganese levels were significantly lower in HE-Zn, HE-Rf and HE rats when compared to controls (*P* = 0.016, *P* < 0.001 and *P* = 0.001, respectively) and significantly higher in HE-Fe, HE-Zn, and HE-Mg rats when compared to HE rats (*P* = 0.007, *P* = 0.046, and *P* < 0.001, respectively) (Supplementary Table 8).

**Figure 5:**
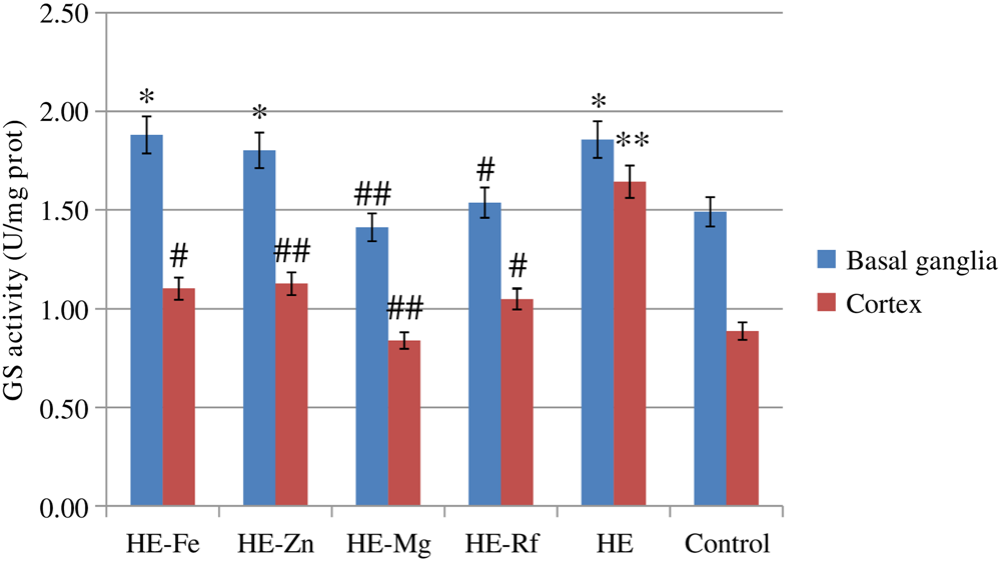
GS activity in the basal ganglia and cortex across groups. A higher basal ganglia GS activity was observed in the HE-Fe rats, HE-Zn rats and HE rats when compared to controls (*P* = 0.036, *P* = 0.045, and *P* = 0.035, respectively). GS activity in the basal ganglia was significantly lower in HE-Mg rats and HE-Rf rats when compared to HE rats (*P* = 0.002 and *P* = 0.038); there were no difference between HE-Mg rats, HE-Rf rats and controls. HE rats had higher cortical GS activity when compared to controls (*P* = 0.003). Significantly lower cortical GS activity was observed in HE-Fe rats, HE-Zn rats, HE-Mg rats, and HE-Rf rats when compared to HE rats (*P* = 0.017, *P* = 0.008, *P* = 0.001, and *P* = 0.016, respectively). Eight rats in each group, *P < 0.05, **P < 0.01, ***P < 0.001, control rat comparison, ^#^ *P* < 0.05, ^##^ *P* < 0.01, ^###^ *P* < 0.001, HE rat comparison. The *P* value is generated using One way ANOVA with a Bonferroni correction.

Higher GS activity was observed in the basal ganglia of HE-Fe, HE-Zn and HE rats relative to controls (*P* = 0.036, *P* = 0.045 and *P* = 0.035, respectively). GS activity in the basal ganglia was significantly lower in HE-Mg and HE-Rf rats when compared to HE rats (*P* = 0.002 and *P* = 0.038). There were no differences in GS activity between HE-Mg, HE-Rf and controls (both *P* > 0.05). HE rats had higher levels of GS activity in the cortex relative to controls (*P* = 0.003). GS activity in the cortex was significantly lower in HE-Fe, HE-Zn, HE-Mg, and HE-Rf when compared to HE rats (*P* = 0.017, *P* = 0.008, *P* = 0.001 and *P* = 0.016, respectively) (Fig. 5, Supplementary Table 9).

## Discussion

In this study, we found that oral administration of iron, zinc and magnesium trace elements, as well as rifaximin, were beneficial to HE rats. Among the four therapies, oral administration of magnesium sulfate was the most effective HE treatment. It significantly reduced the manganese concentration in the brain, regulated ammonia metabolism, decreased GS activity, and consequently, improved cognitive and locomotor impairments in HE rats.

### Effects on manganese concentrations in the brain

HE and HE-Rf rats had significantly decreased fecal manganese excretion and significantly increased manganese concentrations in the brain. These results indicated that rifaximin had no effect on reducing the manganese concentration in the brain. Previous studies reported that decreased zinc and increased copper and manganese concentrations in the brain were related to the development of HE^8, 15^. Zinc has been used to treat HE. Oral zinc treatment could increase fecal manganese excretion and decrease manganese concentrations in the basal ganglia^8^. However, in this study 61 days of treatment with zinc only had a limited effect on decreasing cortical manganese concentrations. One study reported that magnesium could protect against manganese toxicity^16^. However, whether magnesium can be used to treat HE has not been investigated. This study demonstrated that oral magnesium treatment in HE rats significantly decreased manganese concentrations in the basal ganglia and cortex by increasing fecal manganese excretion, which is consistent with previous findings^17^. Iron also increased fecal manganese excretion, which is consistent with previous findings^18^. However, our study showed that iron treatment for 61 days could not effectively reduce cortical manganese concentrations in HE rats.

### Effects on cognition and locomotion

As was reported in previous studies^19, 20^, HE rats exhibited significantly longer escape latencies and a significantly reduced locomotor activity. A previous study reported that zinc could improve cognitive function in patients with HE^12^. In this study, we confirmed that zinc and iron treatments could improve cognition in HE rats. However, cognitive function in HE rats receiving iron or zinc treatment was still lower when compared to controls. Impressively, magnesium treatment significantly decreased escape latencies during the water maze task and increased locomotor activity in HE rats. Previous studies have reported that populations with low magnesium intake had a higher risk of motor neuron disease^21^. Conversely, increases in brain magnesium could improve learning and memory function^21, 22^. In this study, rifaximin also decreased escape latencies and increased locomotor activity in HE rats. These results indicated that ammonia was involved in the cognitive and locomotor impairments reported previously in HE rats^23^.

### Effects on ammonia metabolism

A previous study showed that the non-fasting blood ammonia in rats with partial portal vein ligation gradually decreased in the 2 to 6 week period^24^. This study showed that the fasting blood ammonia levels in HE rats with partial portal vein ligation was in the normal range during the 2 to 8 week period. After glutamate administration, however, the blood ammonia levels in HE rats became significantly higher than in controls. This was thought to be caused by portal-systemic shunting according to previous studies^25, 26^. These results suggest abnormal nitrogen metabolism. Rivera-Mancia S *et al*. suggested that the accumulation of ammonia in the brain was the result of increased manganese concentrations^27^. This study demonstrated that magnesium treatment could significantly reduce blood ammonia levels in HE rats after the administration of glutamate. Magnesium treatment reduced ammonia levels in the blood by increasing fecal manganese excretion which subsequently decreased manganese concentrations in the brain. Similarly, rifaximin treatment also significantly reduced blood ammonia levels in HE rats after the administration of glutamate; however, fecal manganese excretion decreased. Consequently, manganese concentrations in the brain significantly increased. Iron and zinc treatment could not reduce blood ammonia levels in HE rats after glutamate administration. In HE-Fe rats and HE-Zn rats, fecal manganese excretion did not increase. Manganese concentrations in the brain somewhat decreased, but did not decrease to normal levels.

### Effects on GS activity

Studies have confirmed that GS is the key metabolic enzyme in HE^28^. Inhibition of GS activity could improve cognitive function in HE rats^29^. In this study, we found that GS activity significantly increased in the basal ganglia and cortex of HE rats, which was in accordance with a study conducted by Montes S *et al*.^30^. It had been shown previously that ammonia significantly increased GS activity^31^. After rifaximin treatment, significantly lower blood ammonia levels and lower GS activity in the brain were observed. Treatment with iron and zinc slightly decreased GS activity in the cortex, and did not decrease GS activity in the basal ganglia of HE rats. Magnesium treatment significantly reduced GS activity to normal levels in the basal ganglia and cortex. It had been reported previously that manganese and magnesium concentrations effect GS activity^32^. Excessive magnesium can strongly decrease GS activity^32^. Therefore increases in magnesium and decreases in manganese could both cause decreases in GS activity.

This study had some limitations. First, the doses of the trace elements were determined by referring to previous studies. The dose of magnesium sulfate was same as used for mouse models, which exists potential differences from rats. Thus, further studies with different doses should be carried out to evaluate the effects of magnesium sulfate on HE. Second, the doses of ferrum sulfate and zinc sulfate was smaller than that of magnesium sulfate had a limited impact on HE during the 61 day treatment period. Longer treatment durations and higher dose gradients should be investigated. Third, HE-Zn rats had significantly decreased water consumption during the 2 to 8 week period of treatment; its potential effects should be further evaluated.

### Conclusion

In this study, we found that ferrous sulfate, zinc sulfate and magnesium sulfate all significantly reduced the manganese concentration in the brain via increased fecal manganese excretion in HE rats. Among the three trace elements, magnesium sulfate effectively improved cognitive and locomotor impairments in HE rats by significantly reducing the manganese and ammonia concentrations in the brain and decreasing GS activity.

## Methods

### Ethics Statement

This study and the experimental protocols were reviewed and approved by the Institutional Review Board of Jinshan Hospital, Fudan University (2015-05). All procedures were conformed to Institutional Animal Care and Use Committee at the Fudan University guidelines.

### Animal model and treatment

A total of 48 male Sprague-Dawley rats (10 weeks, mean weight 260 g) were used. HE rat models were prepared by partially ligating the portal vein. The surgical procedures are described in a previous report^24^. The HE rats were divided into five groups based on the different treatments administered to each group including three trace elements: HE-Fe group (ferrous sulfate treatment, n = 8), HE-Zn group (zinc sulfate treatment, n = 8), HE-Mg group (magnesium sulfate treatment, n = 8), HE-Rf group (rifaximin treatment, n = 8) served as the positive control, and the HE group (no treatment, n = 8) served at the negative control. Another group consisting of 8 normal rats served as the blank control. The control animals underwent a sham surgery with anesthesia without partially portal vein ligation.

Trace element supplement therapy was administered via drinking water for 61 days. HE-Fe rats were provided ferrous sulfate at a dose of ~81 mg/kg/day (~30 mg/kg/day elemental ferrum)^33^. The dose of ferrum was calculated as follows: (ferrum in feed × food consumption)/water food consumption. HE-Zn rats were provided zinc sulfate at a dose of ~86 mg/kg/day (~30 mg/kg/day elemental zinc)^8^. The zinc dosage was translated from humans to rats as follows: dose for human × (weight of human/weight of rat)^2/3^ ^34^. HE-Mg rats were given magnesium sulfate at a dose of ~107 mg/kg/day (~50 mg/kg/day elemental magnesium)^35^. HE-Rf rats were given rifaximin at a dose of 50 mg/kg/day^23^. These doses were determined by estimating the minimum effective dose in rats, which has been described in previous studies.

To monitor the dose of trace elements and rifaximin, the rats were placed in metabolic cages. The rats were fed and received water ad lib. Weight, water, food consumption and fecal weight were recorded daily. The amount of trace elements and rifaximin required to reach the target dose was calculated based on the water consumption and body weight of each rat. Treatment began immediately after the partial portal vein ligation and lasted 61 days. After the treatment period, the animals were sacrificed. All rats were provided a feed in accordance with the AIN-93 (Purified Diets for Laboratory Rodents) formulation. The feed contained iron (45 mg/kg, ferrous sulfate), zinc (35 mg/kg, zinc sulfate), magnesium (511 mg/kg, magnesium sulfate) and manganese (10 mg/kg, manganese sulfate).

### Behavioral assessment

#### Cognitive behavior

The Morris water maze (MWM) was used to evaluate learning in rats^36^. The maze consisted a circular tank with 180 cm diameter and 50 cm deep. The tank was filled with water (30 cm deep, 24 ± 1 °C). An invisible escape platform was placed 2 cm below the water surface. The tank was divided into four quadrants (LeftTop, RightTop, LeftBottom and RightBottom). The movement trajectories of the rats were recorded using a computer equipped with a camera. The MWM test began on the 9th week (day 57). The rats were trained for 4 consecutive days. The escape latency to reach the platform was recorded. On day 61, the test trial (memory retention test) was performed. During the test trial, the platform was removed and each rat was allowed to explore the pool for 60 s. The total time spent in each quadrant was recorded.

#### Locomotor function

At the end of the 8th week (day 56), the open-field test was used to evaluate locomotor activity. A 40 × 40 × 35 cm black square box was used. The square field (40 × 40 cm) was divided by a 10 × 10 cm grid line. A single touch of the grid line was recorded by the computer as one spontaneous movement. The total number of spontaneous movements during a 30 min period was recorded.

#### Blood ammonia levels and the glutamine challenge test

The glutamine challenge test was administered after the open-field test on the same day. The rats were fasted for 6–8 hours, and received intragastric administration of glutamine (30 mg glutamine dissolved in 5 ml saline). One ml of blood was drawn for testing the concentration of plasma ammonia before and after 30 minutes of the glutamine challenge test (Vitros350, Johnson & NJ, Johnson, USA).

#### Measurement of trace element concentrations

On the same day after the MWM test trial (day 61), the rats were sacrificed via cervical dislocation and then transcardially perfused with cold saline. The brain was removed. The cortex and basal ganglia were separated according to the anatomical atlas of the rat brain. Six rats were selected randomly in each group. The concentrations of calcium, ferrum, zinc, magnetism, copper, and manganese in the cortex and basal ganglia were measured using an inductively coupled plasma (ICP) spectrometer.

#### Enzyme activity assay

The brain sample was weighed and homogenized. Glutamine synthetase (GS) activity was measured using a GS kit in accordance with the instructions (Jiancheng Bioengineering Company, Nanjing, China).

### Statistical analysis

Statistical analyses were performed using SPSS 16.0 for Windows (SPSS Inc., Chicago, IL, USA). One way ANOVA with a Bonferroni correction was used for the escape latency of MWM, locomotor activity, blood ammonia, brain metal content, and GS activity among groups. All values are represented as the mean ± standard deviation. A P value less than 0.05 was considered statistically significant.

## Electronic supplementary material

Supplementery Table

## References

[1] Ferenci P (2002). Hepatic encephalopathy–definition, nomenclature, diagnosis, and quantification: final report of the working party at the 11th World Congresses of Gastroenterology, Vienna, 1998. Hepatology.

[2] Amodio P (2013). The nutritional management of hepatic encephalopathy in patients with cirrhosis: International Society for Hepatic Encephalopathy and Nitrogen Metabolism Consensus. Hepatology.

[3] Tapper EB (2016). Rifaximin for the prevention of readmissions for patients with hepatic encephalopathy - the price is right. Liver Int.

[4] Marano M (2015). Altered metal metabolism in patients with HCV-related cirrhosis and hepatic encephalopathy. Metab Brain Dis.

[5] Rahelic D, Kujundzic M, Romic Z, Brkic K, Petrovecki M (2006). Serum concentration of zinc, copper, manganese and magnesium in patients with liver cirrhosis. Coll Antropol.

[6] Teng L, Zhang J, Dai M, Wang F, Yang H (2015). Correlation between Traditional Chinese Medicine symptom patterns and serum concentration of zinc, iron, copper and magnesium in patients with hepatitis B and associated liver cirrhosis. J Tradit Chin Med.

[7] Zareifar S, Dehghani SM, Rahanjam N, Farahmand FM (2015). Prevalence of Iron deficiency anemia in children with liver cirrhosis: A cross-sectional study. Int J Hematol Oncol Stem Cell Res.

[8] Chavez-Tapia NC (2013). A systematic review and meta-analysis of the use of oral zinc in the treatment of hepatic encephalopathy. Nutr J.

[9] Lopes PJ (2013). Correlation between serum magnesium levels and hepatic encephalopathy in immediate post liver transplantation period. Transplant Proc.

[10] Carpenter KL (2016). Magnetic susceptibility of brain iron is associated with childhood spatial IQ. Neuroimage.

[11] Zou Y (2014). Cognitive function and plasma BDNF levels among manganese-exposed smelters. Occup Environ Med.

[12] Takuma Y, Nouso K, Makino Y, Hayashi M, Takahashi H (2010). Clinical trial: oral zinc in hepatic encephalopathy. Aliment Pharmacol Ther.

[13] Sanchez-Morito N, Planells E, Aranda P, Llopis J (1999). Magnesium-manganese interactions caused by magnesium deficiency in rats. J Am Coll Nutr.

[14] Garcia SJ, Gellein K, Syversen T, Aschner M (2007). Iron deficient and manganese supplemented diets alter metals and transporters in the developing rat brain. Toxicol Sci.

[15] Layrargues GP, Shapcott D, Spahr L, Butterworth RF (1995). Accumulation of manganese and copper in pallidum of cirrhotic patients: role in the pathogenesis of hepatic encephalopathy?. Metab Brain Dis.

[16] Zaidi S (2005). Early biochemical alterations in manganese toxicity: ameliorating effects of magnesium nitrate and vitamins. Ind Health.

[17] Chan AW, Minski MJ, Lim L, Lai JC (1992). Changes in brain regional manganese and magnesium levels during postnatal development: modulations by chronic manganese administration. Metab Brain Dis.

[18] Davis CD, Wolf TL, Greger JL (1992). Varying levels of manganese and iron affect absorption and gut endogenous losses of manganese by rats. J Nutr.

[19] Mendez M (2011). Portosystemic hepatic encephalopathy model shows reversal learning impairment and dysfunction of neural activity in the prefrontal cortex and regions involved in motivated behavior. J Clin Neurosci.

[20] Bruck J (2011). Locomotor impairment and cerebrocortical oxidative stress in portal vein ligated rats *in vivo*. J Hepatol.

[21] Iwami O, Watanabe T, Moon CS, Nakatsuka H, Ikeda M (1994). Motor neuron disease on the Kii Peninsula of Japan: excess manganese intake from food coupled with low magnesium in drinking water as a risk factor. Sci Total Environ.

[22] Slutsky I (2010). Enhancement of learning and memory by elevating brain magnesium. Neuron.

[23] Odena G (2012). Rifaximin, but not growth factor 1, reduces brain edema in cirrhotic rats. World J Gastroenterol.

[24] Eizayaga F (2006). Altered blood-brain barrier permeability in rats with prehepatic portal hypertension turns to normal when portal pressure is lowered. World J Gastroenterol.

[25] Tallis S (2014). Changes in CNS cells in hyperammonemic portal hypertensive rats. J Neurochem.

[26] Chan CC (2012). Portal-systemic collaterals and hepatic encephalopathy. J Chin Med Assoc.

[27] Rivera-Mancia S, Rios C, Montes S (2012). Manganese and ammonia interactions in the brain of cirrhotic rats: effects on brain ammonia metabolism. Neurochem Res.

[28] Rose CF, Verkhratsky A, Parpura V (2013). Astrocyte glutamine synthetase: pivotal in health and disease. Biochem Soc Trans.

[29] Kant D (2014). The effect of glial glutamine synthetase inhibition on recognition and temporal memories in the rat. Neurosci Lett.

[30] Montes S, Alcaraz-Zubeldia M, Muriel P, Rios C (2003). Role of manganese accumulation in increased brain glutamine of the cirrhotic rat. Neurochem Res.

[31] Kanamori K, Ross BD, Kuo EL (1995). Dependence of *in vivo* glutamine synthetase activity on ammonia concentration in rat brain studied by 1H - 15N heteronuclear multiple-quantum coherence-transfer NMR. Biochem J.

[32] Tholey G, Bloch S, Ledig M, Mandel P, Wedler F (1987). Chick brain glutamine synthetase and Mn2+-Mg2+ interactions. Neurochem Res.

[33] Fitsanakis VA (2008). Measuring brain manganese and iron accumulation in rats following 14 weeks of low-dose manganese treatment using atomic absorption spectroscopy and magnetic resonance imaging. Toxicol Sci.

[34] Reagan-Shaw S, Nihal M, Ahmad N (2008). Dose translation from animal to human studies revisited. Faseb J.

[35] Li W (2014). Elevation of brain magnesium prevents synaptic loss and reverses cognitive deficits in Alzheimer’s disease mouse model. MOL BRAIN.

[36] Dhanda S, Sandhir R (2015). Role of dopaminergic and serotonergic neurotransmitters in behavioral alterations observed in rodent model of hepatic encephalopathy. Behav Brain Res.

