# Supplementary material for: Oral administration of trace element magnesium significantly improving the cognition and locomotion in hepatic encephalopathy rats
Source: Sci Rep. 2017 May 12;7:1817. doi: 10.1038/s41598-017-02101-8 (PMC5431966; doi:10.1038/s41598-017-02101-8)
Supplement: Supplementary file 1 — Supplementery Table [file 41598_2017_2101_MOESM1_ESM.pdf]

Oral administration of trace element magnesium significantly improving the cognition and locomotion in hepatic encephalopathy rats

Authors: #Ying LI1, MD., #Chang Xue JI1, MD., #Li Hong MEI2, MD., *Jin Wei QIANG1, MD., PhD., Shuai JU1, MD.,

1. Department of Radiology, Jinshan Hospital, Fudan University, Shanghai 201508, China.

2. Department of Dermatology, Jinshan Hospital, Fudan University, Shanghai 201508, China.

#These authors contributed equally to this work.

* Corresponding Author: Jin Wei QIANG Department of Radiology, Jinshan Hospital Fudan University, Shanghai 201508, China. E-mail: dr.jinweiqiang@163.com.Tel: 0086-21-34189990 Fax: 0086-21-67226910

Additional Information

Competing financial interests: The author(s) declare no competing financial interests.

Author Contributions Statement

Y.L, S.J and CX.J did the animal studies

Y.L and CX.J did the literature research and the drafting of the manuscript

LH.M analyzed and interpreted of data

JW.Q designed study concept and did the critical revision of the manuscript for important intellectual content

All authors reviewed the manuscript

Supplementary table 1 Weight of rats at different time points in different groups

Weight (g)	0w	2w	4w	6w	8w
HE-Fe	256.1±12.2	313.3±12	374±20.7	421.9±28.9	460.6±31
HE-Zn	255.4±18.5	312±20.4	370.3±23.4	418.3±23.7	451.8±23.6
HE-Mg	257.3±17.9	318.5±15	371±21.1	419.3±33.3	458.5±32.2
HE-Rf	266.1±14.4	315.6±8.6	373.6±11.9	422.3±17.8	460.6±20.1
HE	263.5±12.3	310.4±13.6	369.5±15.7	416.6±29	451±28.6
Control	260.4±15.2	311.5±19.2	369.4±14.1	417.1±17.7	452.5±24.9

Eight rats in each group.

Supplementary table 2 Water consumption at different time points in different groups

Water (g/day)	0w	2w	4w	6w	8w
HE-Fe	27.9±4.5	31.6±3.7	35.6±5.1	38.2±3.1	45.2±5.2
HE-Zn	27.3±5	29.8±5.1	30.4±2.7#**	32.2±4.8#**	34.1±5.2###***
HE-Mg	29.9±3.3	32±3.3	34.9±4.6	39.2±5.5	43.5±4.9
HE-Rf	29.5±5.8	30.9±4.2	33.3±1.6	37.4±5.6	43±5.2
HE	28.2±4.6	32.2±4.4	35.4±4.1	38±3.4	44.1±3.5
Control	29.2±4.3	31.8±4.9	36.4±4.5	40.2±4.6	45.8±3

Eight rats in each group, **P*<0.05, ***P*<0.01, ****P*<0.001, compared with control rats, #*P*<0.05, ##*P*<0.01, ###*P*<0.001, compared with HE rats.

Supplementary table 3 Food consumption at different time points in different groups

Food (g/day)	0w	2w	4w	6w	8w
HE-Fe	25.3±2.7	30.2±2	32±2.3	34.1±1.8	38.4±4.2
HE-Zn	25.3±2.1	27.7±2.7	29.9±2.6	32.6±2.3	35.5±4.3
HE-Mg	26.5±2.7	30.1±2	31.7±2.4	35.2±1.7	38.2±2.7
HE-Rf	25.4±1.8	30±1.6	32.5±2.1	35.5±6.8	40.5±4.5
HE	26.5±2.5	29.3±3.5	31.7±2.8	33±2.2	39.4±5.6
Control	26.4±2.2	30.1±3.7	32.6±4.3	35.7±5.5	37.8±5.8

Supplementary table 4 Feces weight at different time points in different groups

Feces (g/day)	0w	2w	4w	6w	8w
HE-Fe	1.78±0.18	1.89±0.33	1.94±0.4	1.94±0.39	2.02±0.35
HE-Zn	1.77±0.2	1.84±0.4	1.86±0.54	1.92±0.76	1.91±0.56
HE-Mg	1.74±0.24	1.8±0.53	1.82±0.55	1.86±0.56	1.95±0.56
HE-Rf	1.75±0.44	1.85±0.32	1.87±0.67	1.9±0.63	2±0.73
HE	1.72±0.29	1.8±0.32	1.85±0.63	1.89±0.57	1.94±0.67
Control	1.77±0.59	1.88±0.29	1.9±0.47	1.9±0.62	1.93±0.52

Eight rats in each group.

Supplementary table 5.1 The escape latency of Morris water maze in training trail

Latency (s)	Test1	Test2	Test3	Test4
HE-Fe	58.1±3.1	47.7±11.3	45.4±13.6**	39.3±14.2***
HE-Zn	57.2±8	51.3±13.8	48±11.4***	40.5±16.5***
HE-Mg	56.4±5.8	44.2±14.7#	36.8±12.4###	26.9±12.5###
HE-Rf	56±6.1	44.9±16.6	39.5±17##	28±9.4###
HE	58.6±4.5	52.5±10.2*	50.4±7.1***	46±14.1***
Control	56.9±6.6	43.3±15.9	34.6±12.6	24.1±12.8

Eight rats in each group, *$P<0.05$, **$P<0.01$, ***$P<0.001$, compared with control rats, #$P<0.05$, ##$P<0.01$, ###$P<0.001$, compared with HE rats.

Supplementary table 5.2 The time spending in the different quadrants of Morris water maze in testing trail

Time (s)	Left Top	Right Top (Target)	Left Bottom (Opposite)	Right Bottom
HE-Fe	11.8±2.9	25.4±3.1###***	11.5±3.4	11.2±2.6
HE-Zn	12.6±2.8	23.1±3.1###***	11.1±2.4	11.5±2.7
HE-Mg	10.3±3.3	31.7±2.8###	8.7±2.6	8.6±2.3

HE-Rf	10.6±2.8	28.1±3.4###**	9.2±2.2	9.8±2
HE	13.3±3.2	19.4±3.4***	13.6±2.9	11.8±3.2
Control	9.4±3	30.4±3.3	9.7±3.2	9.5±2.7

Eight rats in each group, *P<0.05, **P<0.01, ***P<0.001, compared with control rats, #P<0.05, ##P<0.01, ###P<0.001, compared with HE rats.

Supplementary table 6 Locomotor activity at the end of the 8th week in different groups

	Locomotion (times)
HE-Fe	729.6±99.6#
HE-Zn	732.4±123.8#
HE-Mg	742.4±113.5#
HE-Rf	737.4±146.3#
HE	539.8±204.4**
Control	783.9±88.8

Eight rats in each group, *P<0.05, **P<0.01, ***P<0.001, compared with control rats, #P<0.05, ##P<0.01, ###P<0.001, compared with HE rats.

Supplementary table 7 Blood ammonia before and after glutamine challenge in different groups

Blood ammonia (μmol/L)	Base line	Glutamine challenge
HE-Fe	154.4±29.1	440.9±57.3***
HE-Zn	140.8±33.4	424.6±55.6***
HE-Mg	149.6±25.8	234.3±39.1###
HE-Rf	139.5±40.1	242±63.9###
HE	150±24.1	437.8±38***
Control	129.5±19.4	227±94.1

Eight rats in each group, *P<0.05, **P<0.01, ***P<0.001, compared with control rats, #P<0.05, ##P<0.01, ###P<0.001, compared with HE rats.

Supplementary table 8 Trace element concentrations in feces in different groups

Feces	Ca (mg/g)	Mg (mg/g)	Fe (mg/g)	Cu (mg/g)	Zn (mg/g)	Mn (mg/g)
HE-Fe	-	1.44±0.35	0.2±0.05#***	-	0.52±0.26	0.22±0.03##
HE-Zn	-	1.46±0.53	0.11±0.04	-	0.94±0.08***	0.19±0.03#*
HE-Mg	-	1.83±0.43*	0.12±0.02	-	0.49±0.17	0.28±0.02###**
HE-Rf	-	1.48±0.41	0.14±0.04*	-	0.72±0.2*	0.15±0.03***
HE	-	1.58±0.35	0.15±0.04*	-	0.81±0.33*	0.14±0.05**
Control	-	1.28±0.28	0.1±0.03	-	0.43±0.15	0.24±0.02

Six rats in each group, *P<0.05, **P<0.01, ***P<0.001, compared with control rats, #P<0.05, ##P<0.01, ###P<0.001, compared with HE rats, - < 0.01 mg/g.

Supplementary table 9 GS activity in basal ganglia and cortex in different groups

GS activity (U/mg prot)	Basal ganglia	Cortex
HE-Fe	1.88±0.32*	1.1±0.33#
HE-Zn	1.8±0.19*	1.13±0.12##
HE-Mg	1.41±0.19##	0.84±0.32##
HE-Rf	1.54±0.29#	1.05±0.4#
HE	1.86±0.27*	1.64±0.46**
Control	1.49±0.35	0.89±0.37

Eight rats in each group, *P<0.05, **P<0.01, ***P<0.001, compared with control rats, #P<0.05, ##P<0.01, ###P<0.001, compared with HE rats.

AIN-93 Purified Diets for Laboratory Rodents

Components	AIN-93（per kg）
**Calories:**	3601 kCal
Water	6.80%
Fat	4.00%
Carbohydrate	72.70%
Protein	12.50%
Ash	3.89%
**Amino Acid:**	
Alanine	3.3g
Arginine	4.5g
Asparagic acid	8g
Glutamate	25.5g
Glycine	2.3g
Lysine	9.2g
Methionine	3.3g
Cysteine Acid	2.4g
Tryptophan	1.6g
Proline	14.3g
Serine	6.7g
Histidine	3.3g
Leucine	10.9g
Isoleucine	5.9g
Phenylalanine	6.2g
Tyrosine	6.6g
Threonine	4.7g
Valine	7g
**Mineral:**	
Calcium	5000mg
Phosphorus	3000mg
Potassium	3600mg
Sodium	1033mg
Magnesium	511mg
Iron	45mg
Zinc	35mg
Manganese	10mg
Copper	6mg
Iodine	0.2mg
Chromium	1mg
Inorganic sulfur	300mg
Chlorine	1613mg

Vitamin:

Vitamin A	4IU/g
Vitamin D	1IU/g
Vitamin E	0.075IU/g
Vitamin K	0.86mg
Thiamine, B1	5mg
Riboflavin	6mg
Nicotinic Acid	30mg
Pantothenic Acid	15mg
Vitamin B6	6mg
Choline	1000mg
Folic Acid	2mg
Biotin	0.2mg
Vitamin B12	25 μ g
